# Transcriptional Effects of Candidate COVID-19 Treatments on Cardiac Myocytes

**DOI:** 10.3389/fcvm.2022.844441

**Published:** 2022-05-24

**Authors:** Tobias Jakobi, Julia Groß, Lukas Cyganek, Shirin Doroudgar

**Affiliations:** ^1^Department of Internal Medicine and the Translational Cardiovascular Research Center, University of Arizona – College of Medicine – Phoenix, Phoenix, AZ, United States; ^2^Department of Cardiology, Angiology, and Pneumology, Heidelberg University Hospital, Heidelberg, Germany; ^3^DZHK (German Centre for Cardiovascular Research), Partner Site Heidelberg/Mannheim, Heidelberg, Germany; ^4^Stem Cell Unit, Department of Cardiology and Pneumology, University Medical Center Göttingen, Göttingen, Germany; ^5^DZHK (German Centre for Cardiovascular Research), Partner Site Göttingen, Göttingen, Germany

**Keywords:** COVID-19, SARS-CoV-2, chloroquine, hydroxychloroquine, remdesevir, ritonavir, lopinavir, cardiac myocytes

## Abstract

**Introduction:**

Severe acute respiratory syndrome coronavirus 2 (SARS-CoV-2) disease (COVID-19) has emerged as a major cause of morbidity and mortality worldwide, placing unprecedented pressure on healthcare. Cardiomyopathy is described in patients with severe COVID-19 and increasing evidence suggests that cardiovascular involvement portends a high mortality. To facilitate fast development of antiviral interventions, drugs initially developed to treat other diseases are currently being repurposed as COVID-19 treatments. While it has been shown that SARS-CoV-2 invades cells through the angiotensin-converting enzyme 2 receptor (ACE2), the effect of drugs currently repurposed to treat COVID-19 on the heart requires further investigation.

**Methods:**

Human induced pluripotent stem cell-derived cardiac myocytes (hiPSC-CMs) were treated with five repurposed drugs (remdesivir, lopinavir/ritonavir, lopinavir/ritonavir/interferon beta (INF-β), hydroxychloroquine, and chloroquine) and compared with DMSO controls. Transcriptional profiling was performed to identify global changes in gene expression programs.

**Results:**

RNA sequencing of hiPSC-CMs revealed significant changes in gene programs related to calcium handling and the endoplasmic reticulum stress response, most prominently for lopinavir/ritonavir and lopinavir/ritonavir/interferon-beta. The results of the differential gene expression analysis are available for interactive access at https://covid19drugs.jakobilab.org.

**Conclusion:**

Transcriptional profiling in hiPSC-CMs treated with COVID-19 drugs identified unfavorable changes with lopinavir/ritonavir and lopinavir/ritonavir/INF-β in key cardiac gene programs that may negatively affect heart function.

## Introduction

The current COVID-19 pandemic has resulted in more than 271 million confirmed cases as of December 2021 with more than five million deaths reported to be directly linked to the SARS-CoV-2 infection (1). To address the need of treatment options for COVID-19, several different drugs are currently being investigated as possible options. Remdesivir (Rem) is a broad-spectrum antiviral drug initially developed to treat hepatitis C infections. While Rem did not yield the expected results against hepatitis C, it was later tested as treatment option for the Ebola virus (2). Although less efficient against Ebola than monoclonal antibody-based treatments, further trials during an Ebola outbreak from 2013 to 2016 were able to demonstrate its safety (3). With the rise of COVID-19 infections in early 2020, a new study showed effectiveness against severe acute respiratory syndrome (SARS) and Middle East respiratory syndrome (MERS) in animal models (4), thus making it a promising treatment option against SARS-CoV-2. Lopinavir/ritonavir (LR), a combination of lopinavir and ritonavir was specifically developed to treat and prevent the human immunodeficiency virus infection and acquired immune deficiency syndrome (HIV/AIDS) (5). Since *in vitro* studies with SARS and MERS yielded promising results (6, 7), the treatment is being studied either in its original combination LR or as LRI, with the addition of interferon-β (INF-β), since LR and INF-β were earlier shown to improve outcome in a non-human primate model of MERS (8). Chloroquine (CQ) has immunomodulatory effects and is widely used to treat several forms of malaria (9). Hydroxychloroquine (HCQ) was found to be less toxic compared to CQ (10) and is also commonly used for prevention and treatment of malaria as well as other conditions such as lupus or post-Lyme arthritis. Its immunomodulatory effect was proposed to be valuable during the cytokine storm in severely ill patients (9) and recent studies found *in vivo* antiviral activity against SARS-CoV-2 (9, 11).

While the investigations of the repurposed drugs provide rapid insights into current clinical questions, knowledge of how specific cell types react to treatment with the candidate drugs is still scarce. The infection with SARS-CoV-2 is facilitated by binding to the human angiotensin-converting enzyme 2 (ACE2) using the viral surface proteins (12). While ACE2 receptors are highly abundant in lung cells, the receptor is also expressed in the heart (13). Moreover, cardiomyopathy is described in patients with severe COVID-19, and increasing evidence suggests that cardiovascular involvement portends a high mortality (14, 15). Emerging evidence suggests that patients with COVID-19 present with cardiac abnormalities, including new myocardial infarction, myocarditis, and takotsubo cardiomyopathy (16). Moreover, a recent prospective study suggests that even after recovery from a COVID-19 infection, 60% of the patients suffer from ongoing myocardial inflammation independent of preexisting conditions (17), indicating yet-unknown long-term effects of COVID-19 on the cardiovascular system. In this novel global, unbiased study, we integrated molecular, biomedical, and bioinformatics approaches to examine the effects of candidate COVID-19 treatments on human induced pluripotent stem cell-derived cardiac myocytes (hiPSC-CMs). Our findings shed light on the effects of new candidate treatments on molecular pathways and help to assess potential effects and side effects of the treatments.

## Materials and Methods

### Maintenance of Human Induced Pluripotent Stem Cells

Experiments were performed using the human induced pluripotent stem cell (hiPSC) line UMGi014-C clone 14 (isWT1.14) provided by LC (Stem Cell Unit, University Medical Center Göttingen). The iPSC generation and characterization for pluripotency and genomic stability was described previously (18). The line was reprogrammed from somatic cells of a healthy 35-year-old Caucasian male individual. Human iPSC cultures were maintained in Stem MACS iPS Brew XF Medium (Miltenyi Biotec; #130-104-368) in 17 μg/cm^2^ Growth Factor Reduced Matrigel-coated (Corning; #354230) 6-well dishes in a humidified normoxic incubator (37°C, 5% CO_2_). Cultures were routinely passaged in colonies at a ratio of 1:16 every 4–5 days after dissociation using Versene Solution [0.48 mM ethylenediamine tetraacetic acid (EDTA)] (Thermo Fisher; #15040033). Cells were plated in Stem MACS iPS Brew XF Medium and 1 μl/ml of 2 mM Thiazovivin (Millipore; #420220) in DMSO (final concentration 2 μM) for the first 24 h. The culture medium was changed daily with 2 ml per well Stem MACS iPS Brew XF Medium.

### Directed Differentiation of Human Induced Pluripotent Stem Cells Into Ventricular Cardiac Myocytes

HiPSCs were differentiated along the ventricular lineage *via* the modulation of the WNT signaling pathway as previously described (19). Briefly, single cells were harvested using Versene Solution and plated on 6-well dishes at 120,000–160,000 per well into a final volume of 2 ml per well. Differentiation was started when the hiPSC cultures reached a confluency of 80–95% using 3 ml per well Cardio Differentiation Medium [RPMI 1640 (with GlutaMAX and HEPES) (Thermo Fisher; #72400021) supplemented with 0.2 mg/ml L-ascorbic acid 2-phosphate (Sigma; #A8960) and 0.5 mg/ml human recombinant albumin (Sigma; A9731)] and freshly added 4 μM CHIR99021 (Millipore; #361559; 0.4 μl/ml of 10 mM stock solution in DMSO). After 24 h, medium was exchanged to Cardio Differentiation Medium. On day 2, medium was changed to 3 ml Cardio Differentiation Medium supplemented with freshly added 5 μM IWP2 (Millipore; #681671; 1 μl/ml of 5 mM stock solution in DMSO) for 2 days and afterward medium was changed to 3 ml Cardio Differentiation Medium for another 2 days and then again medium was changed to 3 ml Cardio Differentiation Medium for another 2 days. From day 8 onward, medium was changed to 2 or 3 ml of Cardio Culture Medium RPMI 1640 (with GlutaMAX and HEPES) supplemented with the final concentration of 1 × B-27 Supplement (from 50×; Thermo Fisher; #17504044) per well every 2 or 3 days, respectively. On day 15, cells were detached using 0.25% trypsin-EDTA solution (Thermo Fisher; #25200056) and re-plated in 2 ml Cardio Culture Medium supplemented with 20% fetal bovine serum (Thermo Fisher; #10270106, Lot no: 2243865, South American FBS) and 2 μM Thiazovivin at 1 × 10^6^ cells per well in 6-well dishes and medium was changed to Cardio Culture Medium the following day and again on day 18. On day 20, cardiac myocyte selection (20) was performed by changing medium to 2 ml Cardio Selection Medium RPMI 1640 (without glucose and HEPES) with 0.2 mg/ml L-ascorbic acid 2-phosphate and 0.5 mg/ml human recombinant albumin, as well as a final concentration of 4 mM lactate/HEPES (1:250 from 1 M stock) for 2 days. On day 23, medium was replaced with 2 ml Cardio Selection Medium. Starting on day 25, cultures were maintained in Cardio Culture Medium, with regular media changes every 2–3 days. Cells were re-plated after day 30 at 750,000 cells per well in 6-well dishes or at 160,000 cells per well in 24-well dishes for MTT. Experiments were performed on 60-day differentiated cells (hiPSC-CMs).

### Drug Treatment of Human Induced Pluripotent Stem Cell-Derived Cardiac Myocytes

Cultures were treated with or without 5 μM chloroquine (Sigma; #C6628) (21), 5 μM hydroxychloroquine (Sigma; # H0915), 5 μM remdesivir (Biosynth Carbosynth; AG170167), 25 μM lopinavir/ritonavir (Sigma; #SML1222-10MG, #SML0491-10MG), and lopinavir/ritonavir/8 U interferon-β (Sigma; #IF014) for 24 h. After treatment, cells were analyzed as described below. Drug concentrations chosen are based on literature data and are below the 50% cytotoxic concentrations (22, 23). Incubation of cardiac myocytes with drugs for 24 h under normal culture conditions did not result in cytotoxicity.

### MTT Assay

Cell viability was determined using the MTT assay, which measures the reduction of thiazolyl blue tetrazolium bromide (MTT) into an insoluble formazan product by the mitochondria of viable cells. In brief, hiPSC-CMs were seeded into 24-well plates at a density of 160,000 cells per well. The cells were treated with various concentrations of drugs or DMSO as a vehicle control. After 24 h incubation, 50 μl 0.5 mg/ml MTT solution (Sigma-Aldrich; Merck KGaA) was added to each well, followed by further incubation for 4 h. The medium was then removed, and the formazan crystals were dissolved in 500 μl solubilization buffer (10% SDS in 0.01 M HCl). The absorbance was measured at 570 nm on a plate reader (Perkin Elmer, EnSpire Multimode Plate Reader). The relative cell viability was expressed as a percentage of the control group.

### RNA Sequencing

Total RNA was isolated from cultured cells by using QIAzol Lysis Reagent (Qiagen; #79306) according to manufacturer’s instructions. RNA sequencing (RNA-seq) was carried out *via* a commercially available long non-coding RNA service (BGI, Shenzhen, China). Briefly, total RNA was fragmented into short fragments and ribosomal RNA was removed. The cDNA synthesis was performed using random priming. Double-stranded cDNA was purified and enriched by PCR amplification, after which the library products were sequenced using BGISEQ-500.

### Quantitative Real-Time PCR

Total RNA was isolated from cultured cells by using QIAzol Lysis Reagent (Qiagen; #79306) according to manufacturer’s instructions, and reverse-transcribed into complementary DNA (cDNA) by using iScript cDNA Reverse Transcription Kit (Biorad; #1708891). Quantitative real-time PCR was performed using iTAQ SYBR Green PCR Kit (Biorad; #1725124) according to the manufacturer’s instructions.

### Bioinformatics Analysis

Paired-end rRNA-depleted sequencing data were analyzed in detail. After initial quality assessment, low quality regions and adapter sequences were removed with Flexbar (24) (version 3.5). Residual rRNA reads were removed using Bowtie2 with an rRNA sequence-based index (25). Principal read mapping against the ENSEMBL human reference genome build 100 (hg38) was performed with the STAR RNA-seq aligner (26) (version 2.7.5a). Mapped reads were assigned to genes using the Rsubread package (27) (version 2.2.6). Quality of sequencing data and mapping results was assessed with MultiQC (28) (version 1.9). Differential gene expression was analyzed with edgeR (29) (version 3.30.3). Gene Ontology (GO) analyses were performed using topGO version 2.40.0 with all genes having an RPKM ≥ 1 throughout all samples acting as background list. Pathway analyses were performed using the PathVisio software (30) (version 3.3.0) and individual pathways provided by WikiPathways (31). Heatmaps were generated using the ComplexHeatmap package (32) (version 2.5.4). All downstream analyses were performed with R version 3.6.3.

### Statistical Analysis

Cell culture experiments were performed in at least three independent experiments with at least three biological replicates per experiment. Statistical analysis was performed using GraphPad Prism 7.0 (Graphpad Software Inc.^1^) or R. Data values are mean ± standard error of the mean (SEM). For statistical analyses, when only two conditions where compared, unpaired two tailed *t*-test was used.

## Results

### Human Induced Pluripotent Stem Cell-Derived Cardiac Myocytes Treated With COVID-19 Candidate Treatments Show Distinct Gene Expression Patterns

**FIGURE 1:**
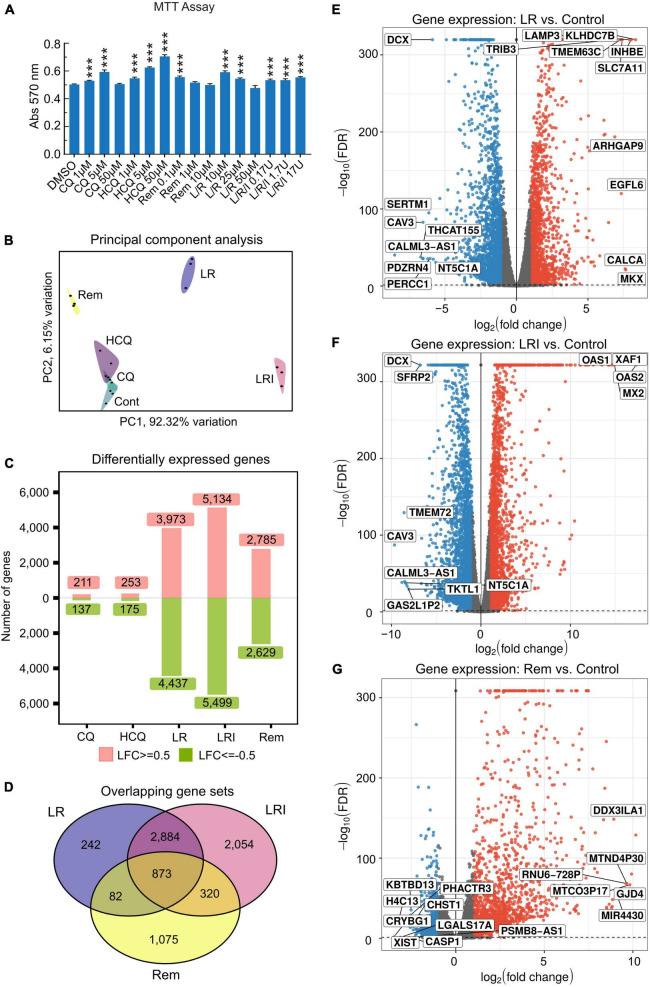
Differences in the response of cardiac myocytes to drug treatments assessed by RNA-seq. **(A)** Various concentrations of each drug were added to hiPSC-CMs for 24 h, and cell viability was determined using the MTT assay. Absorbance at 570 nm was measured using a plate reader. All the cell viability assays were performed at least three times. L/R/I: 25 μm L/R + 0.17, 1.7, or 17U interferon. Two-group comparisons were performed using Student’s two-tailed *t*-test. Data are represented as mean with all error bars indicating ± s.e.m. ***P ≤ 0.001 compared to DMSO control. **(B)** Principal component analysis of RNA-seq data from hiPSC-CMs treated with the five drugs and control samples. CQ (blue): chloroquine; HCQ (light purple): hydroxychloroquine; LR (dark blue): lopinavir/ritonavir; LRI (pink): lopinavir/ritonavir + interferon-β; Rem (yellow): remdesivir. **(C)** Absolute numbers of differentially expressed genes downregulated (green: Log2 fold change ≤ 0.5) and upregulated (red: Log2 fold change ≥ 0.5) after treatments compared to control. **(D)** Shared differentially expressed genes between LR, LRI, and Rem. **(E,F)** Differential expression of genes shown in volcano plots in panel **(E)**, LR vs. control, in panel **(F)**, LRI vs. control, and in panel **(G)**, Rem vs. control. Blue: significantly downregulated, red: significantly upregulated, gray: not significantly expressed, FDR ≤ 0.05.

Incubation of 60-day differentiated hiPSC-CMs with a range of concentrations of the drugs chosen based on literature data for 24 h under normal culture conditions did not result in cytotoxicity as assessed by MTT assay (Figure 1A). Next, hiPSC-CMs were treated with the drugs at selected, relevant experimental doses, which are most commonly used. Total RNA was isolated using QIAzol Lysis Reagent and RNA samples (RNA integrity number [RIN] ≥ 9.6) were subjected to deep sequencing library preparation and RNA sequencing. Principal component analysis of RNA-seq data showed a clear clustering of samples by treatments (Figure 1B). Specifically, samples treated with LR, LRI, and Rem cluster far apart from each other as well as the control samples. Investigation of the differentially expressed genes in each candidate treatment versus vehicle control yielded noticeable differences in the number of differentially expressed genes (Figure 1C). The numbers of differentially expressed genes in CQ and HCQ treatments were comparable with 350–400 genes (FDR ≤ 0.05, |log_2_ fold change| ≥ 0.5) differentially expressed with CQ or HCQ compared to vehicle control. The number of differentially expressed genes was higher by one order of magnitude for LR, LRI, and Rem treatment compared to control; moreover, the number of upregulated and downregulated genes was balanced. We continued to specifically investigate differentially expressed genes shared between LR, LRI, and Rem. On the one hand, overall, nearly 900 genes were shared between the three treatments (Figure 1D). On the other hand, LR and LRI share more than 3,700 genes, with 2,054 differentially regulated genes that were specific to the LRI treatment. The number of genes specific to LR was one order of magnitude less, while around 1,000 differentially expressed genes were specific to Rem. Further inspection of the top differentially expressed genes for LR and LRI showed a strong downregulation of Caveolin-3 (CAV3) that has been implicated in the biogenesis of t-tubules (33) and moreover been shown to be associated with cardiac hypertrophy and heart failure when expression is decreased (34) (Figures 1E,F). On the other side of the spectrum, LR treatment showed a strong upregulation of the transcript of calcitonin gene-related peptide (CALCA), a protein that is secreted from the heart during ischemia or simulated ischemia (35, 36). LRI treatment yielded in upregulation of two genes of the 2′,5′-oligo(A) synthetase family, OAS1 and OAS2 (Figure 1F), which are known to be interferon-inducible (37) and thus is in line with the treatment. Interestingly, one of the top upregulated transcripts after Rem treatment is MIR4430 (Figure 1G), a hardly characterized microRNA that however was recently associated with other repurposed drugs to combat COVID-19 in an *in silico* study (38). A comprehensive list of significantly differentially expressed genes for each comparison is provided in Supplementary Table 1. Moreover, we set up an interactive web portal for visualization of differential gene expression results, which can be accessed *via* https://covid19drugs.jakobilab.org.

**FIGURE 2:**
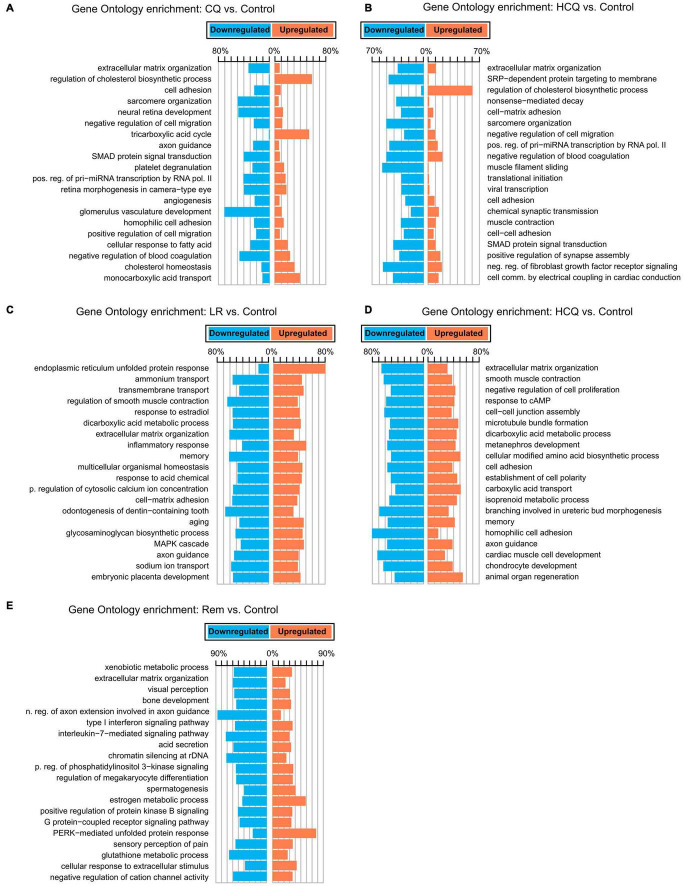
Gene Ontology (GO) functional enrichments for candidate COVID-19 treatments. **(A)** CQ vs. control. **(B)** HCQ vs. control. **(C)** LR vs. control. **(D)** LRI vs. control. **(E)** Rem vs. control. FDR ≤ 0.05. CQ: chloroquine; HCQ: hydroxychloroquine; LR: lopinavir/ritonavir; LRI: lopinavir/ritonavir + interferon-β; Rem: remdesivir.

To gain insights into the biological basis for the differences in gene expression, we performed GO-Term enrichment analyses for each of the differential gene expression experiments and identified key pathways. While the number of differentially expressed genes is lower in CQ and HCQ when compared to LR, LRI, and Rem, pathway analyses showed significant enrichment and upregulation of GO categories of cholesterol biosynthesis, cholesterol homeostasis, and tricarboxylic acid cycle. In contrast, genes in GO categories for sarcomere organization, muscle filament sliding, and cardiac conduction were downregulated with HCQ and in part with CQ treatment (Figures 2A,B). Decrease of gene products in these categories may underlie reduced contractile function. Strikingly, for LR we identified a strong upregulation of the endoplasmic reticulum (ER) unfolded protein response (Figure 2C). During LRI treatment we observed an overall downregulation throughout all enriched GO molecular process categories. Specifically, cardiac muscle cell development and homophilic cell adhesion were strongly downregulated (Figure 2D). However, for both, LR and LRI the enriched GO categories showed both, up and downregulation to comparable extents, while for Rem we observed a more global trend to downregulation in the enriched GO categories, with the notable exception of the PERK-mediated branch of the unfolded protein response, which was strongly upregulated (Figure 2E).

**FIGURE 3:**
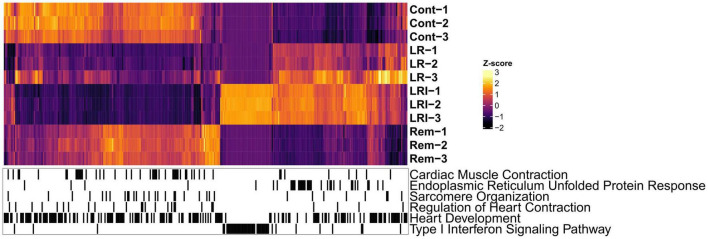
Global map of Gene Ontology (GO) functional enrichments after LR, LRI, and Rem treatment. Heatmap based on TPM-normalized RNA-seq data (orange: high expression, purple: low expression) for differentially expressed genes between control and LR, LRI, or Rem (FDR ≤ 0.05, | Log2 fold change ≥ 0.5|). Below, black lines indicate genes associated with the respective GO category. TPM: Transcripts Per Kilobase Million. LR: lopinavir/ritonavir; LRI: lopinavir/ritonavir + interferon-β; Rem: remdesivir.

Next, we performed hierarchical clustering based on the RNA-seq data from LR, LRI, and Rem treatments, mapped genes involved in these key pathways onto the heatmap, and observed a clear grouping based on treatments (Figure 3). Compellingly, we identified a compact cluster of around 400 genes upregulated in LRI (Figure 3), many of which are associated with type I interferon signaling and thus clearly differentiating between the LR and LRI treatments. Furthermore, we recognized an enrichment for upregulated genes involved in the ER stress response for LR and LRI treatments compared to control. In contrast to the ER stress response genes upregulated with LR and LRI treatments, several other key pathways showed large-scale downregulation of genes such as cardiac muscle contraction, regulation of heart contraction, sarcomere organization, and heart development (Figure 3). While downregulation of genes involved in these pathways was most pronounced with LRI treatment, LR treatment showed a similar trend, although with lower log_2_ fold changes. In comparison to LR and LRI, only few genes of these pathways show downregulation with Rem treatment. Moreover, the log_2_ fold changes of those genes are lower compared to LR and LRI, thus indicating a less significant contribution of Rem treatment to potentially unfavorable changes in these gene programs.

### Dysregulation of Endoplasmic Reticulum Stress and Key Cardiac Function Pathways in LR and LRI-Treated Human Induced Pluripotent Stem Cell-Derived Cardiac Myocytes

**FIGURE 4:**
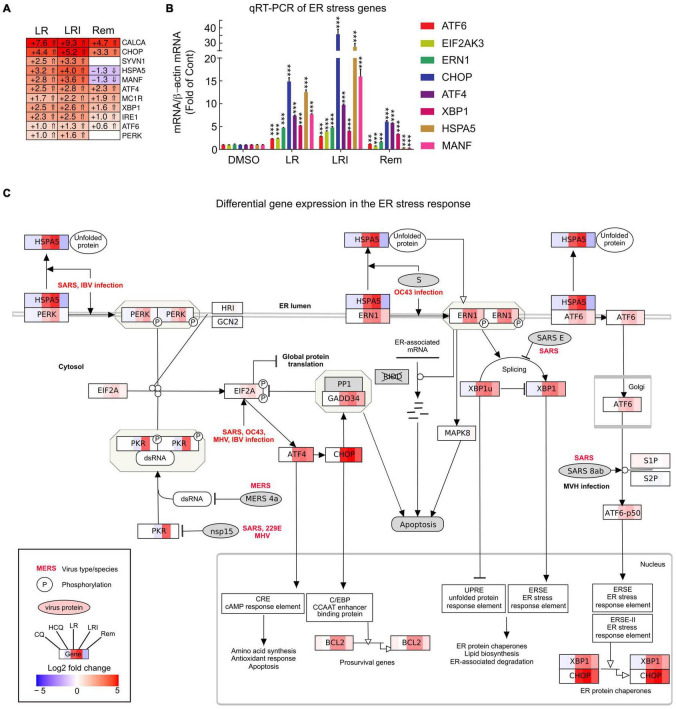
Expression of ER stress associated genes with LR, LRI, and Rem treatment. **(A)** Clustered heatmap of a panel of selected ER stress associated genes showing the calculated Log2 fold change after LR, LRI, and Rem treatment compared to control (FDR ≤ 0.05, | Log2 fold change| ≥ 0.5). LR: lopinavir/ritonavir; LRI: lopinavir/ritonavir + interferon-β; Rem: remdesivir. **(B)** Gene expression was determined by qRT-PCR using RNA from hiPSC-CMs plated at 1 × 10^6^ cells/well on 6-well culture dishes and treated for 24 h with LR, LRI, Rem, or vehicle control (DMSO). **(C)** Mapping of differential gene expression data onto a representation of the ER stress response pathway. Each box contains a color-coded representation of the Log2 fold change of the gene in the following sample order: CQ, HCQ, LR, LRI, Rem. CQ: chloroquine; HCQ: hydroxychloroquine. Two-group comparisons were performed using Student’s two-tailed *t*-test. Data are represented as mean with all error bars indicating ± s.e.m. **P ≤ 0.01 and ***P ≤ 0.001 compared to DMSO control.

To obtain further insights into the details of the altered pathways, we combined differential gene expression results and pathway structures. We performed the pathway analysis using KEGG/wikipathway due to the ability to directly integrate not only the gene names into pathways, but also directly map expression values into the graphical pathway. This way, we were able provide more information within the representative pathway as a Reactome pathway analysis could have provided. We selected a panel of 11 key ER stress response genes and profiled their expression after LR, LRI, and Rem treatment (Figure 4A, FDR ≤ 0.05, log_2_ fold change ≥ 0.5). We observed a strong upregulation of the ER stress response genes for LR and LRI that was less pronounced in Rem-treated samples. Specifically, mesencephalic astrocyte-derived neurotrophic factor (MANF) and heat shock protein family A (Hsp70) member 5 (HSPA5) show significant downregulation after Rem treatment but are upregulated after LR and LRI treatment. To validate our findings, we performed qRT-PCR on a set of eight ER stress response genes and found, in line with the RNA-seq data, upregulation of nodal ER stress response regulators, such as C/EBP homologous protein (CHOP), activating transcription factor 4 (ATF4), X-box binding protein 1 (XBP1), and endoplasmic reticulum to nucleus signaling 1 (ERN1) in LR and LRI compared to control samples (Figure 4B). With Rem treatment, only CHOP, ATF4, and XBP1 showed upregulation, while HSPA5 and MANF were downregulated, which is in line with the RNA-seq data. Furthermore, we overlaid gene expression data with a pathway representation of the ER stress pathway and observed an upregulation of ER stress response genes at several key positions in the pathway, such as XBP1, CHOP, HSPA5, ERN1, ATF6, and PERK (Figure 4C). These results demonstrate dysregulation of the ER stress response with LR and LRI treatments.

**FIGURE 5:**
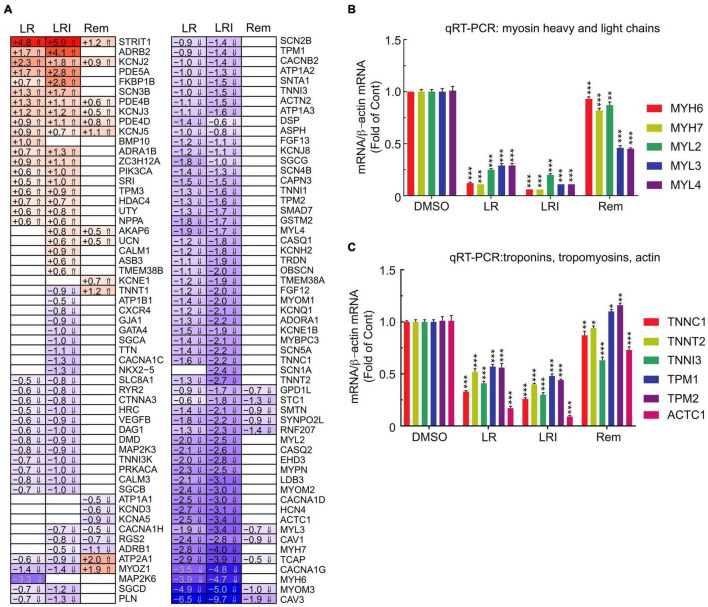
Calcium handling-associated genes and genes associated with sarcomere organization with LR, LRI, and Rem treatment. **(A)** Clustered heatmap of a panel of 100 selected genes associated with calcium handling and sarcomere organization showing the calculated Log2 fold change after LR, LRI, and Rem treatment compared to control (FDR ≤ 0.05, |Log2 fold change| ≥ 0.5). LR: lopinavir/ritonavir; LRI: lopinavir/ritonavir + interferon-β; Rem: remdesivir. **(B)** Expression of myosin light and heavy chains was determined by qRT-PCR using total RNA from hiPSC-CMs plated at 1 × 10^6^ cells/well on 6-well culture dishes and treated for 24 h with LR, LRI, or Rem. **(C)** Expression of selected troponin, tropomyosin, and actin genes was determined by qRT-PCR using total RNA from hiPSC-CMs plated at 1 × 10^6^ cells/well on 6-well culture dishes and treated for 24 h with LR, LRI, or Rem. Two-group comparisons were performed using Student’s two-tailed *t*-test. Data are represented as mean with all error bars indicating ± s.e.m. *P ≤ 0.05, **P ≤ 0.01, and ***P ≤ 0.001 compared to DMSO control.

Our initial global pathway analysis (Figure 3) showed downregulated genes in LR and LRI treated samples that were associated with cardiac muscle contraction, regulation of heart contraction, and sarcomere organization. Based on these GO term associations, we curated a list of 100 genes, profiled their expression in detail (Figure 5A, FDR ≤ 0.05, |log_2_ fold change| ≥ 0.5), and observed that 80% of these genes show decreased expression after treatment with LR and LRI, while only 20% show increased expression. In contrast, only 14 of the 100 selected genes show overall significant differential expression with Rem treatment, including the upregulated troponin T1, slow skeletal type (TNNT1), ATPase sarcoplasmic/endoplasmic reticulum Ca2+ transporting 1 (ATP2A1), and Myozenin-1 (MYOZ1). We validated the RNA-seq data by performing qRT-PCR for two sets of genes, comprising heavy and light myosin chains (Figure 5B). We observed a strong decrease in myosin heavy and light chain transcripts for LR and LRI, whereas the heavy chain transcripts were more strikingly downregulated than the light chain transcripts in LR (Figure 5B). In LRI-treated cells, we observed a strong decrease in expression of both types of myosin chains. In contrast, Rem treatment significantly decreased MYL3 and MYL4, while MYL2 and MYH7 were only slightly, but significantly decreased, and MYH6 levels were unchanged compared to control (Figure 5B).

We subsequently examined a transcript panel of troponins, tropomyosins, and cardiac muscle alpha actin and found a general downregulation after LR and LRI treatment with a striking effect on cardiac actin (ACTC1, Figure 5C). In contrast, Rem treatment resulted in upregulation of tropomyosin 1 and 2 (TPM1, TPM2), while TNNC1 (troponin C1, slow skeletal and cardiac type) and TNNT2 (troponin T2, cardiac type) were slightly but significantly downregulated and ACTC1 and troponin I3, cardiac type (TNNI3) were significantly downregulated compared to vehicle control. These findings suggest significant negative effects of LR and LRI on contractile components of cardiac myocytes.

## Discussion

In this study we evaluated the effects of the five drugs repurposed to treat COVID-19 on hiPSC-CMs. Currently, little is known about the effect of the treatments on different tissues and cells, including cardiac cells. Although there have been studies addressing the effects of treatment with CQ (39), HCQ (40), and LR (41, 42) *in vitro* in different cell lines, to the best of our knowledge no assessment of the effects of CQ, HCQ, LR, LRI, and Rem on cardiac cells has been performed. Here, we provide a detailed analysis of the transcriptional changes in hiPSC-CMs after treatment with the five treatments compared to controls.

### Chloroquine and Hydroxychloroquine Treatments

The anti-malarial activity of CQ is mainly attributed to the diffusion of CQ into lysosomes, which neutralizes the pH and becomes trapped in lysosomes by protonation, thus resulting in the inhibition of normal autophagy activity (43). Moreover, CQ is also used as adjuvant in several cancer therapy trials which employ the autophagy inhibiting properties of CQ. In this context, a recent study revealed a role for cholesterol biosynthesis in maintaining lysosomal integrity under stress; since inhibiting autophagy interferes with processing extracellularly derived cholesterol esters, thus making cells dependent on the cholesterol biosynthesis pathway (44). Moreover, lysosomal membrane cholesterol decreases permeability to water and ions which suppresses swelling and destabilization under osmotic stress. Intriguingly, in our study, we observed that with CQ and HCQ treatment, most of the enriched pathways are downregulated, except for the regulation of the cholesterol biosynthetic process, where CQ- and HCQ-treated samples show strong upregulation for key genes. We found key enzymes for cholesterol synthesis, 3-hydroxy-3-methyl-glutaryl-coenzyme A reductase (HMGCR), hydroxymethylglutaryl-CoA synthase (HMGC1), farnesyl pyrophosphate synthase (FDPS), and farnesyl-diphosphate farnesyltransferase 1 (FDFT1) robustly upregulated with CQ and HCQ treatment. Thus, in line with a previous report (44), our findings may indicate that CQ-treated cells upregulate the cholesterol biosynthesis in order to compensate for reduced processing of cholesterol esters and to counteract CQ-toxicity by adapting the cholesterol biosynthesis pathway. Nevertheless, CQ or HCQ may lead to lower levels of cholesterol, as HCQ was recently found to lower total cholesterol in a large study of patients with systemic lupus erythematosus and rheumatoid arthritis, where CQ and HCQ are common treatment options (45). In contrast, low cholesterol concentrations in COVID-19 patients have recently been linked to more severe outcomes (46, 47). However, due to the short duration of treatment of acute COVID-19 patients compared to long term patients with rheumatoid arthritis treated with CQ or HCQ, the cholesterol-lowering effects might not play a role in progression of COVID-19 in these patients.

The autophagy-inhibiting properties of CQ were found to affect several cellular processes such as bioenergetics (48). Experiments in primary rat cortical neurons showed that the inhibition of autophagy by CQ increased mitochondrial DNA damage and at the same time decreased bioenergetics (48). CQ was found to reduce glycolysis activity, as well as decrease intermediate products of the tricarboxylic acid (TCA) cycle and key components of glutaminolysis (48). In our study, we observed an upregulation of genes enriched for TCA activity with CQ treatment. Indeed, this upregulation may be consistent with an upregulation of glutaminolysis itself as it was previously found that succinate, fumarate, and malate were not affected by CQ-treatment, thus hinting at adaptive changes of the TCA cycle (48). Therefore, our study may contribute to uncovering the transcriptional changes of the TCA cycle components upon treatment with autophagy inhibitors such as CQ or HCQ.

### LR and LRI Treatments

Treatment of hiPSC-CMs with LR or LRI showed significant changes in key cardiac gene programs such as Ca^2+^ handling and sarcomere organization. The downregulation of several key genes encoding subunits of cardiac ion channels we observed such as KCNQ1, KCNH2, and CACNA1C, is in line with findings that show QT prolongation and torsade de pointes in patients treated with protease inhibitors (PI) (49), such as lopinavir and ritonavir. Moreover, the strict downregulation of other key categories of cardiac function such as cardiac muscle cell development with LR and LRI is coordinate with a recent study describing risks of bradycardia with LR treatment for COVID-19 patients (50). Similarly, we observed perturbations in the regulation of PP1, a critical regulator of cardiac function that mediates restoration of contractility to basal levels after beta-adrenergic stimulation. Dysregulation of PP1, in turn, has been suggested to contribute to impaired function of the heart (51), thus highlighting unfavorable effects of LR and LRI treatment on hiPSC-CMs.

Recent guidelines recommend the use of low- to moderate-dose statins for patients with one or more CVD risk factor as preemptive measure (52). However, treatment with statins together with LR or LRI, should be implemented with selected statins such as pitavastatin or pravastatin due to known drug-drug interactions between statins and antiviral PIs (53), thus requiring special attention for COVID-19 patients on statins.

When comparing LR and LRI, we observed specific effects of INF-β as sharply defined enrichment of genes specifically associated in the type I interferon response for LRI. The interferon response employs double-stranded RNA-activated protein kinase (PKR) to reduce viral replication *via* phosphorylation of eIF2α and subsequent reduction of protein synthesis (54). Interestingly, genes induced by INF-β were shown to be associated with ER stress (55) and while in general we observed that the addition of INF-β increased the effects of LR treatment on gene expression, this was especially pronounced for key genes of the ER stress response, such as CHOP, which has been shown to play a role at the interface between ER stress and CVD (56). While the ER stress response exerts an initial protective effect, the strength and duration of activation of the ER stress response will determine if the response will eventually switch to the proapoptotic phase and yield to cell death (56). Alterations in protein folding demand, such as those that occur during cardiac ischemia, hypertrophy, and remodeling, result in homeostatic imbalance in ER, causing activation of the ER stress response and thus induction of translation inhibition and gene expression tailored to stress conditions in the ER (57). We and others have shown that in cardiovascular disease, stresses such as oxidative stress or hypoxia can perturb ER homeostasis and activate the ER stress response, which induces expression of proteins that can function to protect the myocardium (58). Both, the interferon response and ER stress response are known to be activated during viral infection and are linked by the phosphorylation of the α subunit of translation initiation factor eIF-2 (eIF2α) (59, 60), which results in inhibition of synthesis of viral proteins. The upregulation of the ER stress response genes in LR-treated cells, in addition to the ER stress response the viral infection itself, may negatively affect the replication of the virus. This effect might further be enhanced by IFN-β treatment as part of the LRI regime, adding the burden of the activation of the interferon response to the already activated ER stress response. Taken together, our results show elevated expression of key ER stress response genes in LR and even more pronounced in LRI that might result in cell death depending on the duration of LR/LRI treatment.

The activation of the ER stress response caused by PIs was studied in the context of the HIV/AIDS, where PIs are routinely employed as treatment option. While long-term studies for HIV treatment and prevention with LR report general long-term safety (61), cardio-metabolic side effects in the heart have been reported (62). Specifically, lopinavir, which has been shown to induce the highest levels of ER stress amongst PIs (41), may cause adverse effects that have been observed in macrophages (63) and hepatocytes (64). Moreover, clinical studies linked ER stress-associated diseases like metabolic syndrome to patients administered PIs over longer terms (41). Thus, the combination of potential adverse effects and unfavorable dysregulation of key cardiac gene programs after LR and LRI treatment likely outweigh negative effects of LR and LRI on viral infection. Besides dysregulation of critical cardiac gene programs, we observed significant dysregulation of several G protein-coupled receptor-associated (GPCR) genes which in turn might lead to different cardiovascular pathologies, such as hypertrophic and fibrotic remodeling of left and right cardiac ventricles and systemic and pulmonary hypertension (65).

### Remdesivir Treatment

In hiPSC-CMs treated with Rem, we detected a slight, but significant upregulation of some of the ER stress response genes, specifically CHOP, ATF4, and XBP1. Interestingly, we found MANF is significantly downregulated in Rem compared to LR, LRI, and even vehicle-treated control, even though ATF6, a known inducer of MANF (66) is significantly upregulated after Rem treatment. Similarly, HSPA5, a key chaperon in the ER stress response is also significantly downregulated in Rem. Interestingly, HSPA5 which encodes the GRP78 protein, has been identified as a potential target for the treatment of Ebola virus (67) and is currently discussed as a potential treatment for COVID-19 (68, 69). Thus, the downregulation of HSPA5 might be one cause for the more promising results of treating COVID-19 with Rem such as the Adaptive COVID-19 Treatment Trial (ACTT-1) found that patients receiving Rem treatment tend to profit from a significantly shorter time to recovery compared to placebo (70). Available data on the effect of Rem on the heart is limited to a single study of 681 patients infected with the Ebola virus, where one of the patients suffered hypotension and subsequent death by cardiac arrest (71), which might be in line with our observation that several genes associates with hypotension show dysregulation, however, the effect of LR and LRI is more pronounced.

## Conclusion

In summary, the results of this study suggest that all tested repurposed drugs show alterations of the transcriptional profiles of hiPSC-CMs. While the changes to gene programs with CQ and HCQ treatment are less pronounced, we identified widespread unfavorable dysregulation of cardiac gene programs such as calcium handling, sarcomere organization, hypotension and GPCR activity specifically for LR and LRI. The induction of the ER stress response may on the one hand be able to aid in slowing down viral replication, but on the other hand also add adverse effects to the dysregulated gene programs. While treatment of hiPSC-CMs with Rem also induced changes in gene expression, the effect on the cardiac gene programs affected by LR and LRI is significantly more pronounced. Taken together, our results suggest that only Rem displays a fair balance between negative effects on transcriptional profiles of hiPSC-CMs and potential antiviral activity.

## Data Availability Statement

Raw sequencing data are available at the National Center for Biotechnology Information Sequence Read Archive (PRJNA666773). Differential gene expression data is available online in interactive form via https://covid19drugs.jakobilab.org. All other data, methods, and materials that support the findings of this study are available from the corresponding authors on request.

## Author Contributions

TJ and SD: conceptualization, writing–review and editing, and funding acquisition. TJ: data curation, software, writing–original draft preparation, and visualization. TJ, JG, LC and SD: investigation. All authors have read and agreed to the published version of the manuscript.

## Conflict of Interest

The authors declare that the research was conducted in the absence of any commercial or financial relationships that could be construed as a potential conflict of interest.

## Publisher’s Note

All claims expressed in this article are solely those of the authors and do not necessarily represent those of their affiliated organizations, or those of the publisher, the editors and the reviewers. Any product that may be evaluated in this article, or claim that may be made by its manufacturer, is not guaranteed or endorsed by the publisher.

## Footnotes

1
www.graphpad.com

## Supplementary Material

The Supplementary Material for this article can be found online at: https://covid19drugs.jakobilab.org and https://www.frontiersin.org/articles/10.3389/fcvm.2022.844441/full#supplementary-material

Supplementary Table 1 Differential gene expression results as Excel file. CQ vs. Control, HCQ vs. Control, LR vs. Control, LRI vs. Control, and Rem vs. Control.
